# optRF: Optimising random forest stability by determining the optimal number of trees

**DOI:** 10.1186/s12859-025-06097-1

**Published:** 2025-03-31

**Authors:** Thomas M. Lange, Mehmet Gültas, Armin O. Schmitt, Felix Heinrich

**Affiliations:** 1 Breeding Informatics Group, Georg-August University, Margarethe Von Wrangell-Weg 7, 37075 Göttingen, Germany; 2 Faculty of Agriculture, South Westphalia University of Applied Sciences, Lübecker Ring 2, 59494 Soest, Germany; 3 Center for Integrated Breeding Research (Cibreed), Georg-August University, Albrecht-Thaer-Weg 3, 37075 Göttingen, Germany

**Keywords:** Parameter optimisation, Random forest, Machine learning, Non-determinism, Decision-making, Genomic selection, Variable selection

## Abstract

Machine learning is frequently used to make decisions based on big data. Among these techniques, random forest is particularly prominent. Although random forest is known to have many advantages, one aspect that is often overseen is that it is a non-deterministic method that can produce different models using the same input data. This can have severe consequences on decision-making processes. In this study, we introduce a method to quantify the impact of non-determinism on predictions, variable importance estimates, and decisions based on the predictions or variable importance estimates. Our findings demonstrate that increasing the number of trees in random forests enhances the stability in a non-linear way while computation time increases linearly. Consequently, we conclude that there exists an optimal number of trees for any given data set that maximises the stability without unnecessarily increasing the computation time. Based on these findings, we have developed the R package optRF which models the relationship between the number of trees and the stability of random forest, providing recommendations for the optimal number of trees for any given data set.

**Supplementary Information:**

The online version contains supplementary material available at 10.1186/s12859-025-06097-1.

## Introduction

Machine learning is a powerful tool to analyse complex and large data sets and is frequently used across various scientific disciplines in decision-making processes. The term machine learning describes methods that enable the computer to recognise patterns and, in this way, to “learn” from the data and to make predictions for novel data based on the given data [1, 2]. Beyond predictions, these models are increasingly used to make decisions based on their outputs, enabling data-driven decision-making processes [3]. A particularly prominent machine learning method is random forest which is widely used in areas such as finance, healthcare, engineering, and genomic research for data-driven decision-making processes [4–13]. Its popularity is due to the fact that it is a non-parametric method that performs very well, requires very simple input preparation, and can handle binary, categorical, count, and continuous response variables [14].

Random forest works by growing multiple decision or regression trees and making predictions by averaging the predictions of the trees. In order to grow the trees, at each node, the variable is chosen that splits the data set into two distinct groups [15]. Rather than choosing this variable from all available variables, the best variable can be chosen from a random sample of all variables [16]. While some publications state that the value for this parameter, which is typically called *mtry*, can influence the prediction accuracy [17–19], it was also described that the value for *mtry* did not influence the out-of-bag error rate in experiments with microarray data [20]. Next to the number of variables to choose the best variable from, it can also be adjusted how many observations are drawn for the training of each tree. On the one hand, a small value for the sample size can reduce computation time and will lead to more diverse trees which has a positive effect on prediction accuracy. On the other hand, more diverse trees will lead to less stability of the random forest model when aggregating the trees [17]. The depth of the trees can be controlled by defining the minimum number of observations in the terminal node (the node size) [17]. Tuning this hyperparameter can increase prediction accuracy, especially for data sets with a large sample size and a small number of variables [21].

Even though the number of trees grown in a random forest can have a severe effect on the prediction accuracy, this parameter has been described as not tunable because there is no best value for it [17]. While R packages for performing random forest such as randomForest, ranger, and Rborist use as default 500 trees [16, 22, 23], it is widely agreed that a greater number of trees in random forest has only benefits for the quality of the model such as more stable and accurate results without overfitting [24–27]. However, it is also known that the number of trees increases the computation time linearly [17, 27]. This leads to the question of how to set this parameter. It has been recommended to set the number of trees to be ten times the number of variables in the data set and from there to increase the number of trees until the error rate stabilises [25]. Despite the suggestion to set the number of trees as high as the computational power allows it, research with biomedical data sets has shown that from a certain number of trees on, a further increase in the number of trees did not further improve the quality of the random forest model but increased the computation time [28].

One example of data-driven decision-making processes is genomic selection where the phenotype of individuals is predicted using genomic data and subsequently used to select the best individuals in a population where the phenotype is unknown [14, 29]. Such studies rely heavily on the use of single nucleotide polymorphisms (SNPs) which can be analysed in large quantities using SNP chips [30–32]. However, such studies are often limited by the number of individuals that can be analysed, thus, leading to a small number of observations (*n*) and a large number of variables (*p*) which is a general problem in genomic research [33]. While random forest is known to sufficiently work also when the so called “small *n*, large *p*” problem occurs [34–36], it has been discussed that interactions between variables might not be detected and correctly used for predictions when *p* is too large [37–39]. In order to reduce the small *n*, large *p* problem, random forest can be used to estimate the importance of each variable and to select the most important variables from the data set [40–42]. In this way, random forest can also be used to analyse associations between the genotype and the phenotype [43–45]. Doing so, genomic markers can be selected that show the highest association with the phenotype [38, 46–50] and to identify genes related to certain traits [51]. It should be noted that the recommendation to set the number of trees to be ten times the number of variables was not followed in studies regarding genomic selection [11, 52, 53] which could be due to the fact that doing so would require extensive computation time.

Despite the many advantages of random forest, it is often overseen that random forest is a non-deterministic method. This means that repeated runs of random forest with the same data set may lead to different prediction models and varying variable importance estimates. This variability is particularly problematic when the predictions or the estimated variable importances are used in decision-making processes. Even though the impact of non-determinism on decision-making processes may have severe consequences, it has not been adequately addressed to date.

Here, we quantified the stability of random forests with special focus on decision-making processes. Moreover, we analysed the relationship between the number of trees and the stability of the random forest. We analysed this relationship using multiple publicly available data sets from genomic research and show that with all data sets under investigation, the number of trees had a severe effect on the stability of random forest and that this relationship is non-linear. Based on these results, we developed the R package optRF which models this relationship in order to determine the optimal number of trees that leads to a high stability of random forest without unnecessarily increasing the computation time. While it is possible to tune the *mtry* value for random forest using the caret package [54] or the *mtry*, node size, and sample size parameters using the tuneRanger package [17], there is no package available that recommends a number of trees to be set even though we show that regarding the stability of random forest, this parameter is highly important. We fill this void and show that using the optRF package, a certain number of trees is recommended that leads to stable random forest models without unnecessarily increasing the computation time which offers a huge advantage for data-driven decision-making processes based on random forest.

## Materials and methods

### The optRF package

We developed an R package that (I) calculates the stability of a random forest model with *t* trees, (II) models the relationship between number of trees and the stability, and (III) defines an optimal number of trees from where on additional trees would increase computation time but would barely improve the stability further. For this purpose, the R package contains two main functions: opt_prediction to optimise the number of trees for predictions and selection of the best individuals in a test population and opt_importance to optimise the number of trees to estimate the variable importance of each variable and select the most important variables in a data set.

### The opt_prediction function

The opt_prediction function needs as input a training data set where the response variable is inserted in the y argument and the predictor variables are inserted in the X argument. Optionally, the user can specify a test data set using the X_Test argument containing the same predictor variables as in X. The opt_prediction function uses the training data to construct a random forest model with a certain number of trees using the R package ranger [22] and uses this model to predict the response in the test data set. To analyse the data sets described in this paper, we used ranger, version 0.16.0. By default, this process is repeated ten times, leading to a matrix containing ${n}_{test}\bullet 10$ predictions. With this prediction matrix, the prediction stability is calculated and the process is repeated with different numbers of trees, leading to a result table that contains the number of trees and the corresponding prediction stability. By default, opt_prediction calculates the prediction stability for random forests with 250, 500, 750, 1,000, and 2,000 trees.

If the response variable is metric, random forest regression is performed. Therefore, the prediction matrix will contain metric values. Naturally, if the random forest is stable, the repeated predictions will be highly correlated. The prediction stability is, thus, defined as the intraclass correlation coefficient (ICC) [55] between the ten repetitions of random forest. The ICC is calculated using a one-way model and single measures ICC(1,1) [56, 57] using the function icc from the R package irr [58]. If the response variable is categorical, random forest classification is performed and the prediction matrix will contain categorical values. In this case, if the random forest model is stable, the same class will be repeatedly predicted for the same individual in the test data set. Thus, the prediction stability is defined as Fleiss’ kappa (κ) [59, 60] using the function kappam.fleiss from the irr package. For both the ICC and Fleiss’ kappa, a value of 1 would indicate a perfect prediction stability and a value of zero would indicate poor prediction stability [61, 62]. To analyse the data sets described in this paper, we have used irr, version 0.84.1.

To assess the stability of selection decisions based on predicted response values in the test data set, we present the selection stability which is based on a similar metric proposed by Ornella et al. (2014) where the α top performing individuals in the test data set were classified as “selected” once by the observed response values and once by the predicted values and the agreement in these two methods was measured using Cohen’s Kappa [52]. We have defined the selection stability by using the prediction matrix to derive a selection matrix where in each repetition of random forest, the α top performing individuals are also classified as “selected” whereas the remaining individuals are classified as “rejected”. Since ten raters are being compared, we also used Fleiss’ Kappa here, to measure the agreement between these raters. Opposed to the method presented in [52], opt_prediction compares the selection decision based on the ten repeated predicted values in the test data set without the need of knowing the true response values in the test data set.

To evaluate the selection stability when the response is metric, by default opt_prediction selects the 15% individuals with the highest predicted values in the test data set in each repetition of random forest. However, both the number of individuals to be selected as well as the selection criterion can be adjusted by the user. The number of individuals to be selected can be adjusted in the alpha argument of the opt_prediction function. The selection criterion can be adjusted in the select_for argument where either the value “high” (default) can be entered to select individuals with the highest response values, the value “low” can be entered to select individuals with the smallest response values, or the value “zero” can be entered to select individuals where the response is closest to zero. If random forest classification is performed, in each repetition of random forest, individuals are selected for which a certain class or certain classes were predicted. Thus, the user has to specify the name or the names of the desired classes in the select_for argument when using opt_prediction with a categorical response variable.

If no data is provided in the X_Test argument, stability is estimated using the out-of-bag data for each tree grown in the random forest. For a metric response, the prediction for each individual in the data set is defined as the mean of the predictions from the trees in which that individual was not used for training. For a categorical response, the prediction is defined as the class most frequently predicted by the trees where the individual was not included in the training process.

### The opt_importance function

The opt_importance function needs as input the response variable in the y argument and the predictor variables in the X argument. By default, it repeats random forest ten times and in each repetition, the variable importance of each variable is calculated, leading to a $p\bullet 10$ variable importance matrix. If the random forest model is stable, the variable importance estimates will be highly correlated in all repetitions. Hence, the variable importance stability is also defined as the ICC. Also opt_importance calculates the variable importance stability by default with 250, 500, 750, 1,000, and 2,000 trees and derives a result table that contains the number of trees and the corresponding variable importance stability.

Similar to the opt_prediction function, opt_importance also calculates the selection stability. Since variable importance estimates are always continuous, regardless of whether the response is metric or categorical, and higher values indicate more important variables, the selection matrix is derived by classifying the α variables with the highest importance estimates as “selected” and the remaining variables as “rejected” in each repetition of random forest, similar to the approach for classifying variables proposed in [63]. By default, the top 5% of variables are classified as “selected” in each repetition of random forest but this threshold can be adjusted in the alpha argument of the opt_importance function. Selection stability is then quantified using Fleiss’ Kappa which measures the agreement of selected variables across the ten random forest repetitions.

### Modelling the stability

While the function opt_prediction calculates the prediction stability and the selection stability for selecting the best individuals in a test population, opt_importance calculates the variable importance stability and the selection stability for variable selection. In a next step, both functions model the stability (${s}_{j}$) of a random forest model j with ${t}_{j}$ trees with the two parameter logistic (2PL) model

$$\widehat{{s}_{j}}=\frac{1}{1+{\left(\frac{{\theta }_{1}}{{t}_{j}}\right)}^{{\theta }_{2}}}$$

where ${\theta }_{1}$ denotes the number of trees where $\widehat{{s}_{j}}=0.5$ and ${\theta }_{2}$ denotes the slope at ${\theta }_{1}$. This 2PL model equals the three parameter logistic model described in [64] where the maximum effect is set to 1 since the maximum value of the stability measures described above is 1 and the minimum value is 0. The 2PL model can also be derived from the extended four and five parameter logistic models described in [65–67] which have been adapted in multiple studies in the natural sciences [68–70] by setting the asymmetry parameter to 1, assuming no asymmetry within the logistic model. In order to estimate the parameter values, the Levenberg–Marquardt algorithm [71] was applied using the R package minpack.lm [72]. We have used minpack.lm, version 1.2–4, to analyse the data sets described here.

To define an optimal number of trees, the model is used to estimate the increase of stability for each tree being added to the random forest model in the interval from 10 to 10,000,000. Based on the estimated stability per ten trees added to the random forest, the optimal number of trees can be determined. The number of trees will be recommended where an additional ten trees in random forest leads to an increase in stability of 10^–6^ or less. This threshold was set arbitrarily and can be defined by the user in the rec_thresh argument of the opt_prediction and the opt_importance function. By default, opt_prediction will calculate the optimal number of trees based on the prediction stability and opt_importance based on the variable importance stability. However, the user can set the recommendation to be based on the selection stability in both functions.

## Application with real life data

### Data sets

In order to demonstrate the effectiveness of the optRF package, we estimated the optimal number of trees for both genomic selection and for variable selection with 45 publicly available data sets from genomic research (see Table 1 and the Supplementary Table). These data sets were collected from eight different species, namely two data sets from barley (*Hordeum vulgare L.*) [73], one data set from chicken (*Gallus domesticus*) [74] after linkage disequilibrium pruning as in [75], four data sets from maize (*Zea mays* L.) [76], six data sets from rice *(Oryza sativa* L.) [77], eight data sets from rye (*Secale cereale* L.) [78], four data sets from strawberry (*Fragaria × ananassa*) [79, 80], one data set from sugar beet (*Beta vulgaris* L.) [38], and nineteen data sets from wheat (*Triticum aestivum* L.) [81–84]. All data sets exhibited the small *n*, large *p* problem as they contained more variables than observations. The smallest data set, in terms of observations, contained 61 observations and 11,086 variables while the smallest data set, in terms of variables, included 264 observations and 1,134 variables. The largest data set consisted of 1,063 observations and 139,101 variables.

**Table 1:** Analysed data sets with species, response, number of observations (*n*), and number of variables (*p*). For a detailed description of the data sets, see Supplementary Table

Data set	Species	Response	*n*	*p*
1	Barley	Beta-glucan	550	3815
2	Barley	Yield	516	3798
3	Chicken	Egg weight	1063	139,101
4	Maize	Anthesis-silk interval (drought stress)	284	1146
5	Maize	Anthesis-silk interval (well-irrigated)	284	1146
6	Maize	Yield(drought stress)	264	1134
7	Maize	Yield (well-irrigated)	264	1134
8	Rice	Amylose content	386	61,260
9	Rice	Grain yield	937	61,260
10	Rice	*Pyricularia oryzae* infestation	668	61,260
11	Rice	*Nakataea oryzae* infestation	915	61,260
12	Rice	*Rhizoctonia oryzae-sativae* infestation	915	61,260
13	Rice	Yield after milling	937	61,260
14	Rye	Plant height (2018)	572	7718
15	Rye	Plant height (2019)	645	7718
16	Rye	Plant height (2020)	638	7718
17	Rye	Plant height (2021)	572	7718
18	Rye	Yield (2018)	572	7718
19	Rye	Yield (2019)	645	7718
20	Rye	Yield (2020)	638	7718
21	Rye	Yield (2021)	572	7718
22	Strawberry	*Phytophthora cactorum* infestation (2017)	1220	40,313
23	Strawberry	*Phytophthora cactorum* infestation (2018)	1726	40,310
24	Strawberry	*Verticillium dahliae* infestation (2017)	388	34,810
25	Strawberry	*Verticillium dahliae* infestation (2018)	388	34,810
26	Sugar beet	*Beet necrotic yellow vein virus* infestation	156	9127
27	Wheat	Yield (2015)	348	10,560
28	Wheat	Yield (2016)	324	10,560
29	Wheat	Yield (2015, drought stress)	157	10,064
30	Wheat	Yield (2016, drought stress)	150	10,064
31	Wheat	Yield (2017, drought stress)	150	10,064
32	Wheat	Yield (2015, well-irrigated)	157	10,064
33	Wheat	Yield (2016, well-irrigated)	150	10,064
34	Wheat	Yield (2017, well-irrigated)	149	10,064
35	Wheat	Yield (2017)	61	11,089
36	Wheat	Yield (2017)	501	11,089
37	Wheat	Yield (2018)	447	11,089
38	Wheat	Yield (2018)	759	11,089
39	Wheat	Root length	77	9669
40	Wheat	Yield	77	9669
41	Wheat	Germination rate	411	25,200
42	Wheat	*Puccinia striiformis* infestation (2016)	500	25,200
43	Wheat	*Puccinia striiformis* infestation (2017)	500	25,200
44	Wheat	Yield (2016)	500	25,200
45	Wheat	Yield (2017)	498	25,200

All data sets were prepared for the analysis by calculating the mean of the response values if individuals with the same genotype were repeated in the experiment, SNPs with 10% missing values or more or with a minor allele frequency of 1% or less were removed and missing genomic data were imputed with k-nearest neighbours using the function kNN from the R package VIM, version 6.2.2, with default settings [85].

### Detailed analysis of selected data sets

To demonstrate the application of the optRF package, we present detailed results from applying opt_prediction and opt_importance to two example data sets. First, we applied both functions to the maize data set where the yield was measured in well irrigated plots. This is the smallest data set under consideration in terms of variables (264 observations, 1,134 variables, data set 7 in Tab. 1). Second, we applied both functions to the largest data set under consideration where the egg weight was measured in chickens (1,063 observations, 139,101 variables, data set 3 in Tab. 1).

We applied opt_prediction and opt_importance to both example data sets to calculate the prediction and variable importance stability with 250, 500, 750, 1,000, and 2,000 trees, derive the 2PL model described in Eq. 1, estimate the stability for higher numbers of trees, and determine the optimal number of trees. For both example data sets we then calculated the prediction and variable importance stability with larger numbers of trees to show that the model can reliably estimate the prediction and variable importance stability even when derived using only small numbers of trees. Finally, we compared the prediction and selection stability as well as the variable importance and selection stability alongside computation time when performing random forest analysis with 500 trees and with the optimal number of trees for both data sets.

### General application across all data sets

For each data set, we ran opt_prediction once with the argument recommendation set to “prediction” and once with it set to “selection” to determine the optimal number of trees based on the prediction and the selection stability, respectively. These two recommendations were saved and subsequently, random forest was run ten times with default settings of the ranger function which is 500 trees and the prediction and selection stability were calculated. Next, ranger was run ten times with the recommended number of trees for recommendation = “prediction” and the prediction stability was calculated, ranger was run ten times with the recommended number of trees for recommendation = “selection” and the selection stability was calculated, and finally ranger was run ten times with the number of trees defined as ten times the number of variables in the data set as recommended in [25] and the prediction and selection stability were calculated. The same approach was repeated with the opt_importance function where the argument recommendation was set to “importance” to determine the optimal number of trees based on the variable importance stability and to “selection” for selection stability. Subsequently, random forest was again repeated ten times with the default of 500 trees, the optimal number of trees, and with ten times the number of variables as the number of trees. For data set 3, we estimated the stability and computation time with ten times the number of variables as the number of trees to prevent the random forest model from exceeding the available computational power. However, based on the results shown in Fig. 2, we are confident that these estimates are reliable.

All data sets under investigation contained metric response variables. In order to estimate the selection stability, the default settings of opt_prediction were used to select the top 15% individuals and the 5% most important genomic markers with opt_importance. Regarding opt_prediction, the select_for argument was adjusted depending on the response variable. Regarding yield, root length, germination rate, and egg weight, select_for was set to “high”, regarding infestation, β-glucan content, plant height, and amylose content, select_for was set to “low” to select individuals with the lowest values following recommendations in [73, 86, 87], and regarding the anthesis-silk interval, select_for was set to “zero” to select individuals with values closest to zero following recommendations in [52].

## Results

### Results from selected data sets

#### Analysis of the smallest data set

**Fig. 1:**
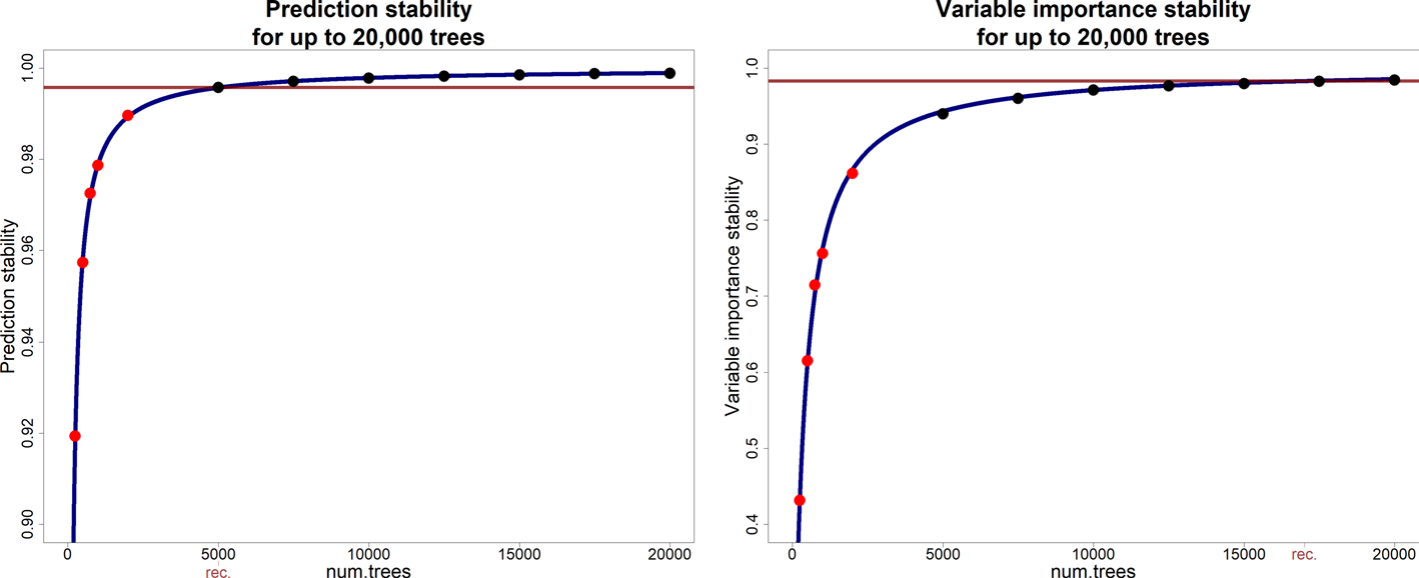
Example of the application of the optRF package to the maize data set (data set 7 in Tab. 1). The relationship between the number of trees and the stability of the random forest was analysed for five numbers of trees (red dots) and estimated using a non-linear model (blue curve) for prediction (left graph) and variable importance stability (right graph). The prediction and variable importance stability with higher number of trees was also calculated (black dots). The horizontal red line indicates the stability with the recommended number of trees (5,000 for prediction and 17,000 for variable importance stability)

The results of applying opt_prediction and opt_importance to the maize data set can be seen in Fig. 1. The red dots represent the stability measures calculated using 250, 500, 750, 1,000, and 2,000 trees, with the relationship modelled using the 2PL model shown as a blue line. The estimated stability at the recommended number of trees is indicated by a horizontal red line. To demonstrate the model’s ability to accurately describe and extrapolate the relationship to higher numbers of trees, the prediction and variable importance stability were calculated using 5,000 to 20,000 trees, shown as black dots in Fig. 1.

One can see in both graphs of Fig. 1 that even though the 2PL model was derived using only the first five data points, the model describes the relationship between the prediction and variable importance stability and number of trees in a random forest accurately which is very important for the computation time of the opt_prediction and the opt_importance function.

In this example, the opt_prediction function recommended to increase the number of trees in random forest by a factor of ten from the default of 500 trees to 5,000 trees with which the prediction stability increases from 0.958 to 0.996. Naturally, this increase in the number of trees would also increase the computation time by a factor of ten. However, one can see in the left graph of Fig. 1 that with 500 trees, the prediction stability increases steeply with each additional tree being added to the random forest while with 5,000 trees, the stability reaches a plateau from where on adding more trees would barely increase the prediction stability further. When selecting the 15% top performing individuals based on the predicted response, the selection stability with the default value of 500 trees is 0.828 while the selection stability with 5,000 trees is at 0.957. In a further analysis, we repeated random forest ten times with 500 and with 5,000 trees and found that 57.5% of the selected individuals were selected in each repetition when random forest was performed with 500 trees and 90% of the selected individuals were selected in each repetition when random forest was performed with 5,000 trees. This demonstrates that a slight increase in prediction stability increased the selection stability markedly.

The opt_importance function recommended for this data set to use random forest with 17,000 trees which increases the number of trees by a factor of 34 compared to the default of 500 trees and, thus, also increases the computation time by this factor. With the default of 500 trees, random forest leads to a variable importance stability of 0.612 which is increased to 0.983 with 17,000 trees. One can see in the right graph of Fig. 1 that also here, the default of 500 trees leads to a part of the model where increasing the number of trees has a strong effect on the variable importance stability. The recommended number of trees is set to be at the part from where on the model flattens and a further increase in the number of trees would barely increase the variable importance stability further. When performing random forest with 500 trees, a selection stability of 0.492 is reached. When selecting the 5% most important variables in ten repetitions of random forest with 500 trees, we found that 14% of the selected variables were selected in each of these repetitions. With the recommended 17,000 trees, a selection stability of 0.918 is reached. When selecting the 5% most important variables in ten repetitions of random forest with this number of trees, 73.7% of the selected variables were selected in each repetition of random forest. Here, the increase in the number of trees strongly affected both the variable importance and the selection stability.

#### Analysis of the largest data set

**Fig. 2:**
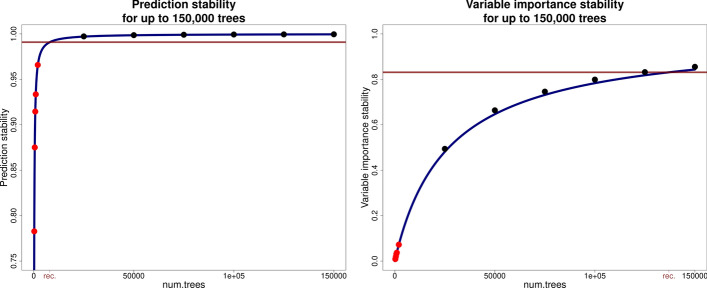
Example of the application of the optRF package to the chicken data set (data set 3 in Tab. 1). The relationship between the number of trees and the stability of the random forest was analysed for five numbers of trees (red dots) and estimated using a non-linear model (blue curve) for prediction (left graph) and variable importance stability (right graph). The prediction and variable importance stability with higher number of trees was also calculated (black dots). The horizontal red line indicates the stability with the recommended number of trees (8,000 for prediction and 137,000 for variable importance stability)

The results from applying the optRF package to the largest data set (egg weight in chicken, data set 3 in Tab. 1) can be seen in Fig. 2. Here as well, the red dots visualise the calculated stability with 250, 500, 750, 1,000, and 2,000 trees and the blue line shows the model that describes the relationship between number of trees and stability for any number of trees. To show that the model can accurately predict the stability for higher numbers of trees, the prediction and variable importance stability were calculated when using 25,000 to 150,000 trees, visualised as black dots. Furthermore, the estimated stability with the recommended number of trees is shown as horizontal red line.

In this example, the functions of the optRF package were applied to the largest data set under consideration and a larger number of trees was necessary to build stable random forest models. But even though the 2PL model was derived with only small numbers of trees, it could accurately estimate the prediction and variable importance stability with up to 150,000 trees. This shows the reliability of the stability estimates from the 2PL model.

Here, the prediction stability was 0.876 when using random forest with the default of 500 trees. The opt_prediction function recommended to increase the number of trees to 8,000 which led to a prediction stability of 0.991. Also here, with 500 trees, the prediction stability increases steeply with each tree being added to random forest while with the recommended number of trees, the prediction stability reaches a plateau from where on adding more trees to random forest barely increases the prediction stability further. When selecting the 15% top performing individuals, the selection stability increases from 0.612 with 500 trees to 0.926 when random forest is performed with 8,000 trees. When repeating random forest ten times, only 30.2% of the selected individuals are selected in these ten repetitions with 500 trees while 82.4% of the selected individuals are selected in each of the ten repetitions of random forest with 8,000 trees.

Regarding the variable importance stability, one can see a dramatic increase in stability when the number of trees was increased from the default of 500 trees. With the default of 500 trees, only a variable importance stability of 0.018 could be reached. The opt_importance function recommended to use 137,000 trees which increased the variable importance stability to 0.845. While this is still deviating from a stability of 1, this is the number of trees from where on adding more trees to the random forest would increase the variable importance stability by 10^–6^ or less and is, thus, a compromise between stability and computation time. When selecting the 5% most important variables from the data set, random forest provides a selection stability of 0.029 with 500 trees which is increased to 0.482 with the recommended 137,000 trees. When repeating random forest ten times with 500 trees, not a single variable was selected in each of the ten repetitions which shows the instability of random forest with only 500 trees for variable selection with this data set. With the recommended number of trees, 19.3% of selected variables were selected in each of the ten repetitions. With this data set, one can see the severe necessity to increase the number of trees for reliable variable selection using random forest.

### General results across all data sets

To show the effectiveness of the optRF package, we applied both opt_prediction and opt_importance to 43 further data sets with various different response variables, numbers of observations, and numbers of variables. For each data set, we calculated the optimal number of trees to select the top performing individuals based on their predicted response values or to select the most important variables. The data sets and the recommended numbers of trees as well as the resulting stability and computation time are given in the Supplementary Table. While we could in general observe that a high number of trees is necessary to build stable random forest models in data sets with a large number of variables, the results also show that different numbers of trees were recommended for data sets with similar numbers of variables. For example, data sets 35 and 38 contain the same response for the same species with the same number of variables. However, data set 35 contains 61 observations while data set 38 contains 759 observations. While 75,000 trees optimise the variable importance stability in data set 35, 9,000 trees are already optimising the variable importance stability in data set 38. This indicates that not only the number of variables but also the number of observations in the data set affects the optimal number of trees.

In the same way, data sets 43 and 44 can be compared. Both data sets analyse the same species with the same number of variables and the same number of observations. However, while data set 43 contains data about the infestation with *Puccinia striiformis*, data set 44 contains yield data. While the variable importance stability was optimised for data set 43 with 18,000 trees, 53,000 trees were necessary to optimise the variable importance stability for data set 44. This indicates that not only the dimensionality of the data affects the recommended number of trees but also the response variable.

**Fig. 3:**
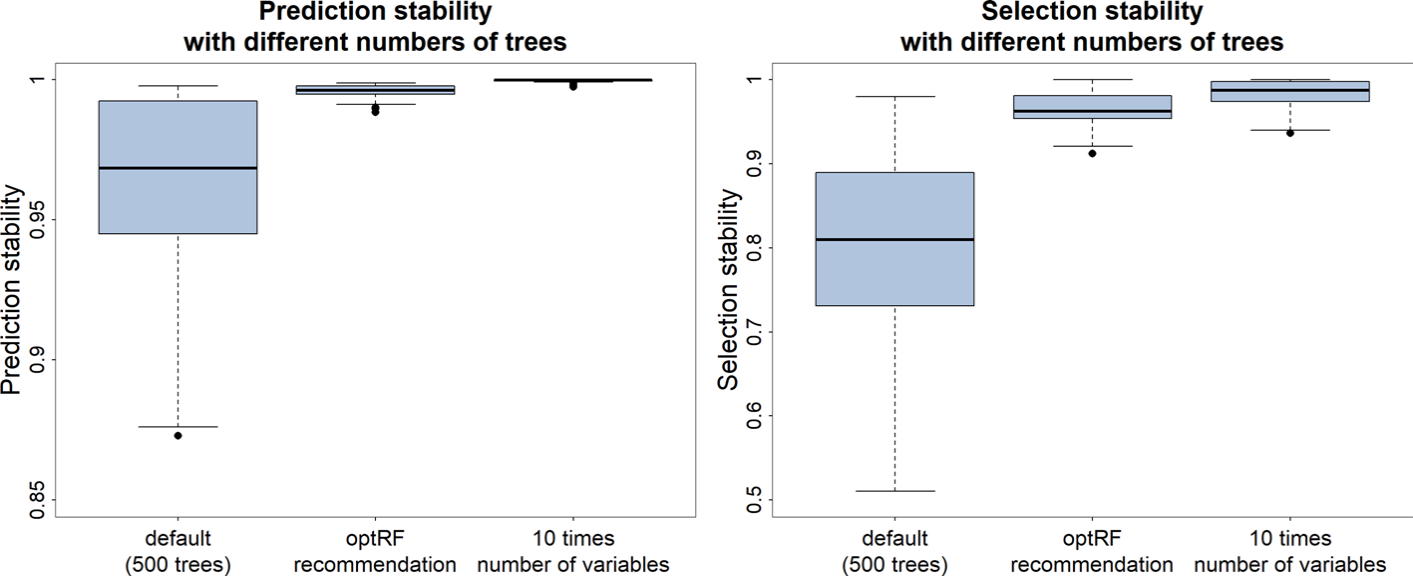
The prediction stability (left graph) and the selection stability (right graph) for all 45 data sets under investigation when using random forest for predictions and to select top performing individuals with (I) 500 trees, (II) the recommended number of trees from the optRF package, and (III) ten times the number of variables as the number of trees

Figure 3 visualises the prediction and selection stability of random forest to select the top performing individuals based on their predicted response values with the 45 data sets under investigation with 500 trees which is the default setting in the ranger function, the recommended number of trees from the optRF package, and the number of trees being ten times the number of variables. One can see in Fig. 3 that both the prediction and selection stability increased markedly when the number of trees was increased from 500 to the optimal number of trees as recommended by the opt_prediction function. The average prediction stability increased from 0.9545 to 0.9957 and the average selection stability increased from 0.7934 to 0.964. Furthermore, one can see that the random forests where the number of trees was set to ten times the number of variables increased the prediction and selection stability only slightly compared to the random forests with the optimal number of trees. The random forests with ten times the number of variables led to an average prediction stability of 0.9996 and an average selection stability of 0.9825.

**Fig. 4:**
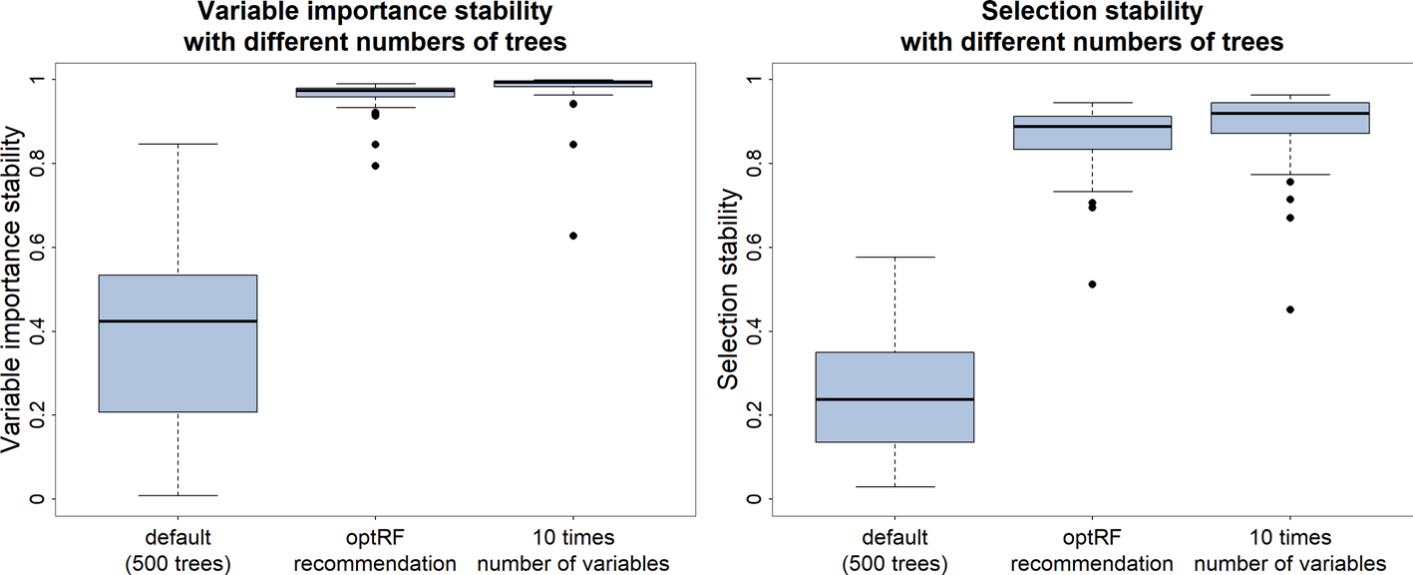
The variable importance stability (left graph) and the selection stability (right graph) when using random forest to select the most important variables with (I) 500 trees, (II) the recommended number of trees from the optRF package, and (III) ten times the number of variables as the number of trees

Figure 4 visualises the variable importance and selection stability of random forest used for variable selection with the 45 data sets under investigation. Here as well, the number of trees was defined as 500, as the recommended number of trees from the opt_importance function, and as ten times the number of variables. One can see that the variable importance stability and the selection stability increased markedly when the number of trees was increased from 500 to the optimal number of trees. Here, the variable importance stability increased from an average of 0.3763 to an average of 0.9563 and the selection stability increased from an average of 0.2533 to 0.8602. The variable importance and selection stability increased in most cases again only slightly when the number of trees was ten times the number of variables. While the variable importance stability increased on average to 0.9688, the selection stability increased on average to 0.8787.

**Fig. 5:**
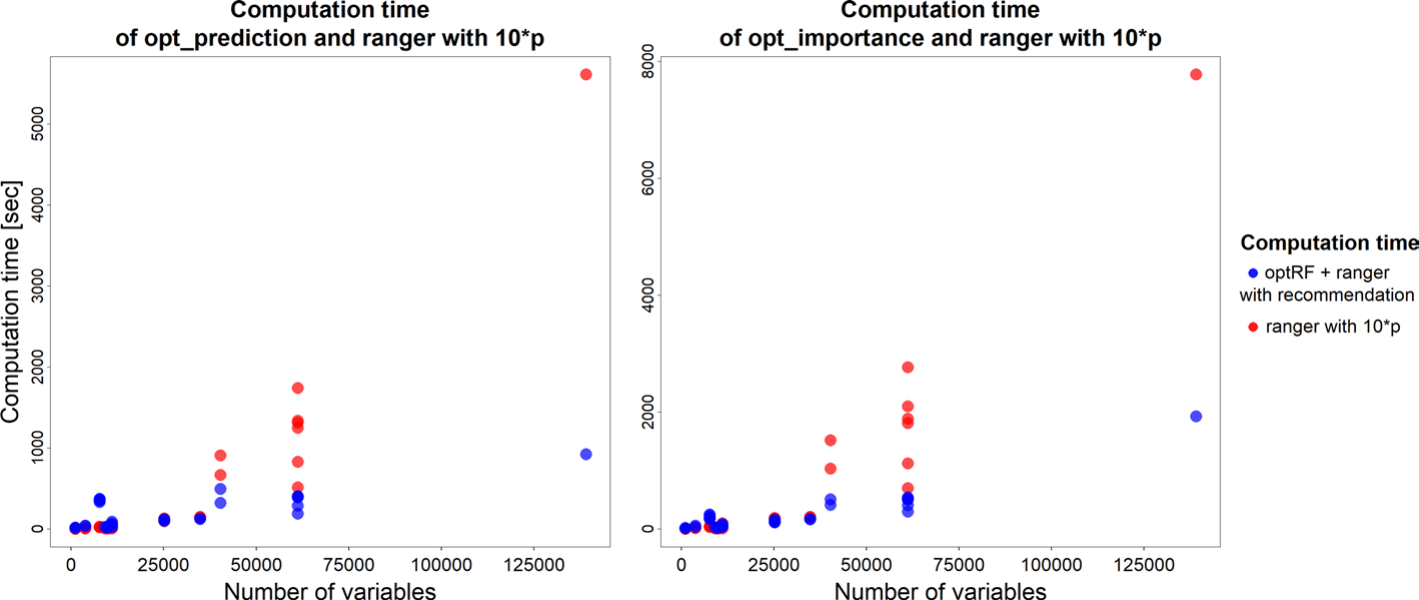
The computation time of the opt_prediction function (left graph) and the opt_importance function (right graph) together with the ranger function run with the recommended number of trees as blue dots compared to the computation time of the ranger function run with ten times the number of variables as the number of trees as red dots for all data sets under investigation with the corresponding number of variables

Next to the stability, we were also interested in the computation time of the different methods to set the number of trees in random forest. Since the default value of 500 trees led to insufficient stabilities, we only focused on comparing the computation time of the optimal number of trees and ten times the number of variables as the number of trees. Therefore, the computation time of the optimal number of trees was defined as the computation time of the opt_prediction or the opt_importance function to determine the optimal number of trees plus the computation time of ranger which was performed with the optimal number of trees. Figure 5 visualises the number of variables in the data sets on the X axis and the corresponding computation time for random forest with the optimal number of trees as blue dots and with ten times the number of variables in the data set as red dots.

As shown in Fig. 5, for data sets with fewer than 30,000 variables, executing opt_prediction or opt_importance followed by running the ranger function with the recommended number of trees required mostly comparable computation time as running ranger with ten times the number of variables as the number of trees. However, for data sets exceeding 30,000 variables, applying opt_prediction or opt_importance in combination with ranger using the recommended number of trees resulted in lower computation time compared to using ranger with ten times the number of variables as the number of trees. These results indicate that the optRF package reduces computation time for data sets with a large number of variables compared to a fixed approach of using ten times the number of variables as the number of trees.

Furthermore, setting the number of trees to be ten times the number of variables still led in some cases to unstable random forests. For example, the second data set had 3,798 variables but showed a variable importance stability of 0.845 and a variable selection stability of 0.671 when random forest was performed with 37,980 trees. The opt_importance function recommended here to use 96,000 trees as the optimal number of trees regarding the variable importance stability and even 708,000 trees as the optimal number of trees regarding the selection stability. This shows that setting the number of trees to be ten times the number of variables can still lead to unstable random forest models. Regarding the stability, the authors actually stated to use random forest with ten times the number of variables as the number of trees and to increase the number of trees further until the error rate stabilises [25]. Thus, the computation time of this approach can be assumed to exceed the approach of the optRF package for most data sets.

## Discussion

Random forest is a useful tool for data-driven decision-making processes. However, in order to fully exploit the potential of random forest, optimal hyperparameters must be set. While it is generally agreed that the number of trees is an extremely important parameter in random forest, it has been argued that this parameter cannot be tuned since there is no value that would maximise the quality of the random forest [17]. Instead, it was generally recognised that the quality of the random forest increases with higher numbers of trees and it was, thus, recommended to use as many trees as the computational power allows. However, we showed that the stability of random forest increases non-linearly with higher numbers of trees while the computation time increases linearly. Thus, it is possible to determine the optimal number of trees that increases the stability of random forest until a further increase of the number of trees only leads to a negligible increase of the stability.

With all data sets under investigation, it could be shown that increasing the number of trees had a strong effect on the prediction and variable importance stability which also increased the selection stability for both the selection of individuals and variables. However, one could also see that this effect was stronger for variable selection than for the selection of the top performing individuals with the data sets under investigation. However, all data sets under investigation had in common that the number of variables exceeded the number of individuals.

In all data sets, the number of trees recommended by the optRF package improved stability markedly. However, considering Fig. 2, the optimal number of trees recommended by the optRF package led to a variable importance stability below 0.9. This is because we defined the optimal number of trees to be the number at which an increase of ten additional trees would increase the stability by less than 10^–6^. While we found this threshold to be adequate, it can be adjusted by the user via the rec_thresh argument in both opt_prediction and opt_importance. Alternatively, opt_prediction and opt_importance can be used to study the relationship between the number of trees and the stability and subsequently, the functions estimate_stability and estimate_numtrees can be used to either analyse the stability of a random forest model with a certain number of trees or to determine the smallest number of trees to achieve a desired level of stability. Moreover, these functions also estimate the computation time, enabling the user to adjust the criteria for determining the optimal number of trees as desired.

When running opt_prediction and opt_importance across all data sets, we found that the optimal number of trees did not only depend on the number of variables in the data set but also on the number of observations and the response under investigation. We found that a higher number of observations leads to a smaller number of trees being necessary for stable variable selection. Moreover, we found that the optimal number of trees required for stable random forest models also depends on the internal structure of the data. In cases where the response is influenced by many weak predictors, a larger number of trees is needed to achieve stability. Conversely, when the response is driven by a few strong predictors, stability can be reached with fewer trees. A large number of trees is also required in cases where the most influential predictors are missing from the data set. When strong predictors exist in reality but are not captured in the available variables, the model relies on many weakly associated variables instead. As a result, more trees are needed to achieve stable variable selection.

One important feature of the opt_prediction function is that the prediction stability is analysed, not the prediction accuracy. While it is necessary for the calculation of the prediction accuracy to compare the predicted values to the observed values in the test data set, the observed values of the test data set do not need to be known to calculate the prediction stability. Thus, in a realistic scenario where the predictor variables in the test population are known but the response is unknown, this method can still be applied. However, this method is most appropriate when a specific test data set is available. Here, we used data sets that did not allow for an evaluation in a scenario where predictions were made for an independent test data set. Although it would have been possible to split each data set into, for example 80% training and 20% test data, such an approach would have reduced the number of observations in the training data set and would have introduced additional stochasticity which could have affected the results. Instead, the opt_prediction function was designed such that, if no test data are provided, the entire data set is used for training and predictions are generated only from trees in which the given individual was not included during training. This approach ensures that opt_prediction provides a realistic estimation of prediction and selection stability for a test data set with a structure similar to that of the training data set.

Despite the many advantages of optimised hyperparameters on the quality of random forest, determining optimal parameter values can be a computational burden [27]. We have developed a method where the relationship between the stability and the number of trees in random forest is modelled with small numbers of trees and with this model, the relationship is extrapolated for higher numbers of trees. Doing so, the computation time to determine the optimal number of trees can be reduced immensely. However, even with this method, the optRF package still needs to calculate the stability with 250, 500, 750, 1,000, and 2,000 trees which requires random forest to be run ten times with each of these numbers of trees. Consequently, for data sets with smaller numbers of variables, running opt_prediction or opt_importance and subsequently running ranger with the recommended number of trees led to a comparable computation time as running ranger with ten times the number of variables as the number of trees. However, when applying this method to data sets with more than 30,000 variables, the computation time was reduced when using the optRF package. Since the computation time of random forest and the recommendation of the optRF package depends not only on the number of variables, the threshold of 30,000 variables from where on optRF performs faster than ten times the number of variables as the number of trees can vary for different data sets. Furthermore, the real advantage of using the optRF package is that the stability can be estimated. In some cases even ten times the number of variables did not result in stable random forest models which would always give reason for doubt. The optRF package on the other hand can offer a measure of how reliable decisions based on the random forest model are.

Although modelling the relationship between stability and the number of trees offers computational advantages, it is based on parametric statistical modelling which requires assumptions to be made regarding the relationship and residual distributions. Furthermore, this process depends on the data used to build the statistical model. Thus, the numbers of trees that are analysed could theoretically have an impact on the recommendation. That is why we give the user the possibility to enter any set of values for the numbers of trees that should be analysed and used to derive the statistical model. Nevertheless, since the recommendations are based on the model’s relationship between the numbers of trees and stability, they can still be influenced by randomness. Therefore, the process of estimating prediction stability and variable importance stability should be repeated and averaged to achieve stable results. We found that repeating the process ten times for each number of trees provides stable estimates but users can increase this number to further stabilise results at the cost of additional computation time. However, with the 45 data sets under investigation, the default settings of the optRF package provided stable recommendations.

It was shown in the results that although the model was derived with only small numbers of trees, it could reliably estimate the prediction and variable importance stability for higher numbers of trees. We observed a similar pattern for selection stability when using the opt_prediction function, however, we cannot assume that this applies universally to all data sets or response variables. In contrast, we observed that opt_importance sometimes under- or overestimated the selection stability for higher numbers of trees. We recommend that the number of selected variables in the alpha argument of opt_importance should approximate the actual number of important variables in the data set to ensure accurate selection stability estimates for higher numbers of trees. As a default setting in the opt_importance function, we set alpha to be 0.05, thus, assuming that 5% of the variables in the data set are truly important, a common assumption in genomic research [88]. While this default is suitable for the data sets analysed here, we advise adjusting alpha for applications outside of genomic research. For variable selection, we suggest first running random forest with a sufficient number of trees (at least equal to the number of variables) to identify highly important variables. Then, the opt_importance function can be used with an adjusted alpha value based on this number.

While R packages such as caret or tuneRanger recommend specific values for *mtry*, the node size, or the sample size [17, 54], optRF provides recommendations for the optimal number of trees. Additionally, whereas caret and tuneRanger focus on maximising prediction accuracy, optRF optimises the number of trees based on the stability of the random forest. Another key distinction is that caret and tuneRanger tune hyperparameters within the training data set, whereas optRF determines the optimal number of trees for both a given training and test data set. Since the optRF package optimises a hyperparameter that is not tuned by the other packages, they can be effortlessly combined to set values for *mtry*, sample size, node size, and the number of trees. However, Probst et al. (2019) pointed out that reducing the sample size can improve prediction accuracy but may come at the cost of decreased stability [17]. As the tuneRanger package selects hyperparameters that maximise accuracy, this may decrease stability. Therefore, we recommend to first optimise hyperparameters such as *mtry*, sample size, and node size and then optimise the number of trees using the optRF package to ensure both accuracy and stability.

Moreover, the functions in the optRF package cannot only be used to determine the optimal number of trees but also to analyse the stability of a random forest model with a certain number of trees. Since the stability of random forest is crucial for the reproducibility of results, we highly recommend to state the number of trees and the stability of the random forest model either as prediction and selection stability or as variable importance and selection stability when publishing results that were determined using a random forest model.

## Conclusion

The results presented here show that the number of trees has an important effect on the stability of random forest. Furthermore, it shows that a random forest model with the default setting of 500 trees provides too much instability for decision-making processes in all data sets that were analysed. Moreover, the results indicate that the number of trees necessary to reach a stable random forest model depends not only on the number of variables but also on the number of observations and internal structures in the data set.

While other R packages aim to maximise the prediction accuracy by tuning *mtry*, the sample size, and the node size, we present a method that optimises the stability of random forest by determining the optimal number of trees. We share the optRF package via CRAN and GitHub to enable others to either search for an optimal number of trees when using random forest for decision-making processes or to estimate the stability of the random forest model with a given number of trees. Since the stability of random forest is crucial for the reproducibility of results, we highly recommend to increase the number of trees until stable results can be retrieved from random forest and to publish the number of trees as well as the stability of the random forest model whenever random forest is used for prediction, variable importance estimation, or decision-making processes.

## Supplementary Information

Additional file 1.

Additional file 2.

## Data Availability

The raw data used in this study are publicly available (see citations in the Materials and methods section). The stability measures and computation time when applying random forest with 500 trees, with the optimised numbers of trees, and with ten times the number of variables as the number of trees are given in the Supplementary Data. R scripts to repeat the analysis with the raw data and the visualisation of the graphs with the results are also given in the Supplementary Data. The optRF package is freely available at CRAN (https://cran.r-project.org/web/packages/optRF/index.html) and at GitHub (https://github.com/tmlange/optRF).

## Declarations

Ethics approval and consent to participate.

Not applicable.
